# 6-Hydroxyflavone Attenuates Inflammatory Osteolysis by Inhibiting Osteoclast Activation via the Nrf2 and Calcium Signaling Pathways

**DOI:** 10.1007/s10753-026-02500-y

**Published:** 2026-04-10

**Authors:** Kun Qian, Qizhen Lu, Weiyi Wang, Qige Lu, Shenghong Dai, Chuanyun Fu, Shui Sun

**Affiliations:** 1 Department of Joint Surgery, Shandong Provincial Hospital Affiliated to Shandong First Medical University, Jinan, Shandong China; 2 Skeletal Aging Research Laboratory, Medical Science and Technology Innovation Center, Shandong First Medical University & Shandong Academy of Medical Sciences, Jinan, Shandong China; 3 Department of Orthopedics, Shanghai Tenth People’s Hospital, School of Medicine, Tongji University, Shanghai, 200072 China; 4 Department of Stomatology, Shandong Provincial Hospital Affiliated to Shandong First Medical University, Jinan, Shandong 250021 China; 5 School of Stomatology, Shandong First Medical University & Shandong Academy of Medical Sciences, Jinan, Shandong 250117 China

**Keywords:** 6-Hydroxyflavone, Inflammatory osteolysis, Osteoclastogenesis, Calcium oscillation, Oxidative stress

## Abstract

Inflammatory osteolysis (IO) is a pathological bone disorder characterized by excessive osteoclast activation and bone resorption driven by inflammatory mediators and oxidative stress. 6-Hydroxyflavone (6-HOF), a natural flavonoid with potent antioxidant and anti-inflammatory properties, has not been fully investigated in the context of IO. In this study, we explored the therapeutic effects and underlying mechanisms of 6-HOF in LPS-induced osteoclastogenesis and inflammatory bone loss. Network pharmacology analysis predicted that 6-HOF primarily targets oxidative stress and calcium signaling pathways. In vitro, 6-HOF inhibited multinucleated osteoclast formation in a concentration-dependent manner without affecting cell viability, downregulated osteoclast-specific genes including *Nfatc1*, *Ctsk*, *Dc-stamp*, and *Mmp9*, and significantly reduced the expression and secretion of pro-inflammatory cytokines IL-6, TNF-α, and IL-1β. Mechanistically, 6-HOF suppressed intracellular ROS accumulation, disrupted RANKL-induced Ca²⁺ oscillations, and inhibited NFATc1 signaling essential for osteoclast differentiation, while activating the Keap1/Nrf2 antioxidant pathway to restore redox homeostasis. In vivo, micro-CT analyses showed that 6-HOF treatment alleviated LPS-induced bone loss by reducing osteoclast numbers and preserving trabecular microarchitecture. Collectively, these results indicate that 6-HOF inhibits osteoclastogenesis and inflammatory responses through the dual regulation of oxidative stress, calcium signaling, and pro-inflammatory cytokine production, highlighting its potential as a promising therapeutic candidate for IO and other bone-destructive disorders.

**Supplementary Information:**

The online version contains supplementary material available at 10.1007/s10753-026-02500-y.

## Introduction

Inflammatory osteolysis is a pathological process characterized by inflammation-mediated overactivation of osteoclasts and progressive bone destruction [1, 2]. The core features of this condition include aberrant osteoclastic activity and enhanced bone resorption [3].It is frequently associated with periprosthetic osteolysis, chronic periodontitis, and rheumatoid arthritis [4–6]. Clinical manifestations encompass persistent bone pain, joint dysfunction, and pathological fractures, significantly impairing quality of life [4, 7, 8]. The pathogenesis involves multiple factors, such as bacterial lipopolysaccharides (LPS) and implant-derived wear particles, which activate the immune system and induce macrophages to release pro-inflammatory cytokines, including TNF-α, IL-1β, and IL-6 [1].These cytokines promote osteoclast differentiation and bone resorption through both RANKL-dependent and RANKL-independent pathways [3, 9].Current treatments, including nonsteroidal anti-inflammatory drugs (NSAIDs), bisphosphonates, and biologic agents, aim to alleviate symptoms, inhibit bone resorption, or target specific inflammatory mediators [10–12]. However, their efficacy is limited by side effects, drug resistance, and high costs. Therefore, developing mechanism-based therapeutic strategies to effectively slow disease progression is of critical importance.

LPS is a classical inducer of inflammatory osteolysis and is widely used to mimic osteoclast activation under inflammation [13]. LPS stimulation strongly promotes osteoclast differentiation and bone resorption [14]. Calcium oscillation and oxidative stress play key roles in this process. Calcium signaling maintains transcription factor activity, while excessive ROS accelerate osteoclast maturation [15, 16].To resist oxidative stress, cells activate the Keap1/Nrf2 pathway. After separating from Keap1, Nrf2 moves into the nucleus and promotes the expression of antioxidant enzymes such as HO-1 and NQO1, which help remove extra ROS [15, 17].Activating Nrf2 or blocking calcium oscillation can effectively reduce osteoclast activity, providing a theoretical basis for preventing LPS-induced osteolysis [15, 18, 19].

Flavonoids have attracted considerable attention in medical research due to their well-established antioxidant and anti-inflammatory properties [20–22]. 6-HOF is a naturally occurring flavonoid monomer widely distributed in vegetables, fruits, and various medicinal plants. Its phenolic hydroxyl group confers good structural stability and pronounced capacity of free radical scavenging, thereby endowing 6-HOF with defined antioxidant activity [20, 23, 24]. Previous studies have demonstrated that 6-HOF exhibits favorable biosafety and low toxicity, and exerts significant anti-inflammatory effects by suppressing inducible nitric oxide synthase (iNOS) and multiple pro-inflammatory mediators [24, 25]. Moreover, 6-HOF alleviates oxidative stress–related injury through scavenging ROS and enhancing antioxidant enzyme activity, showing protective effects in mesangial cells as well as in drug- or chemically-induced liver injury [25, 26]. Notably, osteoclast differentiation and activation are regulated by NF-κB and MAPK signaling, RANKL-dependent Ca²⁺ signaling, and ROS, which intersect with the established anti-inflammatory and antioxidant properties of 6-HOF. Although several flavonoids, such as quercetin and naringenin, have been reported to modulate bone metabolism, no systematic studies have directly examined the role of 6-HOF in osteoclast activity, bone resorption, or inflammatory osteolysis [27].

In recent years, the advancement of network pharmacology and systems biology has provided powerful methodological support for elucidating the multi-target mechanisms of natural compounds [28]. In this study, network pharmacology was first employed to predict the potential targets and signaling pathways of 6-HOF associated with inflammatory osteolysis, followed by in vitro and in vivo experiments to verify its regulatory effects and underlying mechanisms on LPS-induced osteoclastogenesis. Based on this strategy, the present study aims to elucidate how 6-HOF suppresses osteoclast activity by modulating oxidative stress and calcium oscillation pathways, thereby alleviating inflammatory osteolysis and providing theoretical and experimental evidence for the development of natural anti-osteolytic agents.

## Methods and Materials

### Bioinformatics Data Sources and Network Analysis

Potential targets of 6-HOF associated with osteolytic diseases were systematically identified through integrated bioinformatics analysis. Disease-related genes were retrieved from the GeneCards (https://www.genecards.org/) and OMIM (https://www.omim.org/) databases using the keyword *“Osteolysis”* [29]. The two-dimensional MOL structure of 6-HOF was obtained from ChemicalBook (https://www.chemicalbook.com/), and its putative targets were predicted using the SwissTargetPrediction platform (https://www.swisstargetprediction.ch/).

The predicted compound targets and disease-associated genes were intersected using the Bioinformatics Venn tool (https://bioinformatics.psb.ugent.be/webtools/Venn/) to identify overlapping targets. Functional enrichment of the shared targets was performed using the DAVID database (https://david.ncifcrf.gov/) for Gene Ontology (GO) annotation and Kyoto Encyclopedia of Genes and Genomes (KEGG) pathway analysis, with parameters set to *Homo sapiens* and identifiers defined as *Official Gene Symbol* [30]. Visualization of enrichment data was completed using GraphPad Prism 9.0. The complete bioinformatics analysis pipeline is described in detail in Supplementary Material 1.

To elucidate potential interactions among the common targets, the intersected genes were submitted to the STRING database (https://string-db.org/) to construct a protein–protein interaction (PPI) network. The resulting network was imported into Cytoscape 3.10.0 for topological assessment and graphical visualization of key nodes and hub genes.

### Drug-Likeness Prediction and Pharmacokinetic Profiling of 6-HOF

To systematically evaluate the drug-like properties and pharmacokinetic characteristics of 6-HOF, the SwissADME platform (https://www.swissadme.ch/) was employed for comprehensive computational analysis. Based on Lipinski’s “Rule of Five” criteria, the platform calculated key physicochemical descriptors, including molecular weight, lipophilicity (LogP), and hydrogen bond donors/acceptors, to assess oral drug-likeness [31]. ADME (Absorption, Distribution, Metabolism, and Excretion) parameters were further analyzed to predict intestinal absorption, plasma protein binding, metabolic stability, and clearance characteristics [32]. Bioavailability radar visualization was used to quantify six physicochemical dimensions—lipophilicity, size, polarity, solubility, saturation, and flexibility—providing an integrated overview of molecular suitability for drug development. Additionally, the BOILED-Egg model was applied to simulate passive diffusion and active transport by correlating polar surface area (PSA) with lipophilicity (WLOGP), enabling prediction of gastrointestinal absorption and blood–brain barrier permeability [33].

### Reagents and Materials

Cell culture reagents were purchased from Gibco (USA), including fetal bovine serum (FBS; 10099–141 C), minimum essential medium α (α-MEM; C12571500BT), and penicillin/streptomycin (P/S; 15140-122). Reagents for cellular assays were obtained from multiple commercial sources: cytokines M-CSF (576406) and RANKL (769406) were from BioLegend; the small molecule compound 6-HOF (HY-N7110) was provided by MCE; lipopolysaccharide (L8274) and dimethyl sulfoxide (D2650) were sourced from Sigma-Aldrich. TRAP staining reagents were supplied by different manufacturers: sodium tartrate dibasic dihydrate (6106-24-7) and sodium acetate trihydrate (6131-90-4) were obtained from Sigma-Aldrich; Fast Red Violet LB salt (32348-81-5) was supplied by In vivoChem; naphthol AS-TR phosphate disodium salt (4264-93-1) was purchased from Sigma-Aldrich. In addition, the fluorescent calcium indicator Fluo-4 (20551) was acquired from AAT Bioquest, and all isotonic solution reagents were procured from Solarbio. Antibodies for Western blot analysis were sourced as follows: β-actin (sc-47778) antibody was provided by Santa Cruz; Nfatc1 (A1539) was supplied by ABclonal; Ctsk (11239-1AP), Keap1 (10503-2AP), and Nrf2 (16396-1-AP) antibodies were obtained from Proteintech. All secondary antibodies, including anti-rabbit (SA00001-2) and anti-mouse (SA00001-1), were uniformly purchased from Proteintech.

### Cell Culture and Osteoclast Differentiation

Cell culture and osteoclast differentiation were performed based on our previous study with minor modifications [34]. Bone marrow–derived monocytes/macrophages (BMMs) were isolated from tibiae and femur of 8–12-week-old male C57BL/6J mice under sterile conditions. After removal of the epiphyses, bone marrow cells were flushed out with α-MEM, gently dispersed, filtered through a 70-µm cell strainer, and centrifuged. The cells were then seeded in complete medium (CM; α-MEM supplemented with 10% FBS and 1% penicillin–streptomycin) and incubated at 37 °C in a humidified atmosphere containing 5% CO₂ for 16–24 h. Non-adherent cells were collected and treated with red blood cell lysis buffer (ABS-BL503A, Biosharp) for 2 min to remove erythrocytes, followed by centrifugation to obtain BMMs. BMMs were then seeded at a density of 2 × 10⁵ cells/mL and cultured in CM containing macrophage colony-stimulating factor (M-CSF, 10 ng/mL) for 2–3 days for expansion.

Osteoclast differentiation was induced by first culturing cells in CM containing M-CSF (10 ng/mL) and receptor activator of nuclear factor-κB ligand (RANKL, 30 ng/mL) for 3 days to generate osteoclast precursors, followed by another 3 days of either continued M-CSF/RANKL stimulation for functional assays or stimulation with M-CSF (10 ng/mL) and LPS to establish an inflammatory osteoclastogenesis model. To evaluate the effects of 6-HOF on osteoclast differentiation, 6-HOF was added simultaneously with the initial RANKL stimulation in both the RANKL + 6-HOF and LPS + 6-HOF (differentiation) groups and maintained throughout the entire period. To study the effects of 6-HOF after the onset of inflammatory stimulation or during osteoclast activity, we established the LPS + 6-HOF (activity) group, and 6-HOF was administered concurrently with the second LPS stimulation following the initial LPS exposure. Similarly, in the RANKL + 6-HOF (activity) group, 6-HOF was added at the same time point as in the LPS + 6-HOF (activity) group, approximately equal to RANKL stimulation at day 5 to evaluate its effects on mature osteoclast activity.

### Cell Viability Assay

The cytotoxicity of 6-HOF on BMMs was determined using the Cell Counting Kit-8 (CCK-8; Dojindo). BMMs were pre-cultured in α-MEM containing M-CSF (10 ng/mL) for 16–24 h and then seeded into 96-well plates. Cells were treated for 24 h with various concentrations of 6-HOF (0, 5, 10, 15, 20, 25 µM) in complete medium containing either M-CSF alone (10 ng/mL) or M-CSF plus RANKL (30 ng/mL). Absorbance at 450 nm was measured using a Spark^®^ multimode microplate reader (TECAN, Switzerland). Cell viability was calculated as:

[(A_{sample} – A_{blank}) / (A_{control} – A_{blank})]

The relative absorbance values reflected the effect of 6-HOF on BMM viability under osteoclastogenic or basal culture conditions.

### Tartrate-Resistant Acid Phosphatase (TRAP) Staining

TRAP staining was optimized based on our previous protocol [35, 36]. Briefly, cells were washed with PBS and fixed with 4% paraformaldehyde for 15–20 min at room temperature. After rinsing with distilled water, a freshly prepared TRAP working solution was used, containing equal volumes of naphthol AS-TR phosphate disodium (2 mg/mL) and Fast Violet B salt (7 mg/mL) in acetate–tartrate buffer. Samples were incubated at 37 °C for 2 h in the dark, followed by washing with freshly prepared sodium fluoride solution (4.2 mg/mL). TRAP-positive multinucleated cells containing ≥ 3 nuclei were defined as mature osteoclasts and counted under an optical microscope for quantitative analysis.

### F-Actin Ring Staining

F-actin staining was performed following our previous study [37]. Mature osteoclasts were rinsed with PBS and fixed with 4% paraformaldehyde for 15–20 min, followed by ddH₂O washing. Phalloidin–iFluor 594 (Abcam, ab176757) was diluted in PBS containing 0.1% BSA and applied for 2 h of dark incubation at 37 °C. After three PBS washes, nuclei were counterstained with DAPI for 5 min. Images were captured using a confocal fluorescence microscope (Invitrogen EVOS M7000). Osteoclasts containing ≥ 3 nuclei and positive for TRAP were considered mature, and the ratio of F-actin–positive cells to total osteoclasts was calculated to evaluate cytoskeletal organization.

### Acridine Orange (AO) Staining

AO staining was conducted following a modified version of our previous protocol [34].On day 5 of osteoclast differentiation, cells were incubated with prewarmed acridine orange (AO) working solution (10 µg/mL in α-MEM; Sigma-Aldrich, A6014) for 15 min at 37 °C in the dark. After removal of the staining solution, cells were rinsed twice with α-MEM and replenished with fresh medium. Fluorescence images were immediately acquired using a confocal fluorescence microscope (Invitrogen EVOS M7000) under GFP (Ex/Em = 470/525 nm) and RFP (Ex/Em = 531/593 nm) channels.

Fluorescence intensity was quantified using ImageJ. Images were split into red and green channels (“Image → Color → Split Channels”), and osteoclasts were selected as regions of interest (ROIs) using the ROI Manager. Background fluorescence was measured from background regions and used for background correction. Net red and green fluorescence intensities were calculated after background subtraction, and the red/green mean gray value ratio was used to assess osteoclast acid-secretion capacity. Data from all images were pooled for quantitative analysis of AO staining.

### Intracellular Calcium Oscillation Measurement

Intracellular Ca²⁺ oscillations were measured following an optimized protocol [18]. BMMs were seeded onto confocal dishes and cultured with M-CSF (10 ng/mL) for 2–3 days, then induced with M-CSF (10 ng/mL) + RANKL (30 ng/mL) to generate pre-osteoclasts. Treatment groups were established as follows: LPS and 6-HOF groups were switched to medium containing M-CSF and LPS; RANKL and RANKL + 6-HOF groups remained in the original medium for an additional 12–24 h.

Fluo-4 AM stock (1 mM) was prepared in F127/DMSO solution (0.2 g F127 per 1 mL DMSO) and diluted in complete medium to a working concentration of 5 µM under light-excluded conditions. Cells were incubated with the probe for 50 min in the dark, washed twice with ISO buffer (105 mM NaCl, 5 mM KCl, 6 mM HEPES, 4 mM Na-HEPES, 5 mM NaHCO₃, 60 mM D-mannitol, 5 mM D-glucose, 1.3 mM CaCl₂, 0.5 mM MgCl₂; pH 7.4), and returned to fresh ISO. Time-lapse imaging was performed on a confocal microscope (Invitrogen EVOS M7000) at 5-s intervals for 20 min. Fluorescence dynamics in ten selected cells were analyzed with ImageJ; oscillation amplitude was expressed as F/F₀ based on the initial fluorescence.

### Intracellular ROS Detection

Intracellular ROS levels were measured using the DCFH-DA fluorescent probe (Beyotime, China), following the manufacturer’s protocol. BMMs were induced with M-CSF (10 ng/mL) and RANKL (30 ng/mL) for 3 days to obtain pre-osteoclasts, then treated with LPS (100 ng/mL) and/or 6-HOF for 24 h. Cells were washed twice with PBS and incubated with 10 µM DCFH-DA in serum-free α-MEM for 30 min at 37 °C in the dark. After incubation, cells were washed three times with PBS to remove unbound dye. Fluorescence images were captured using a fluorescence microscope (Invitrogen EVOS M7000) under the FITC channel (Ex/Em = 488/525 nm). The mean fluorescence intensity (MFI) was quantified using ImageJ software to evaluate ROS generation in each group.

### Reverse Transcription Quantitative PCR (RT-qPCR)

Total RNA was extracted from cultured cells using RNAiso Plus (9109, Takara) according to the manufacturer’s protocol. Reverse transcription was performed with the PrimeScript RT Kit (RR047A, Takara) to synthesize cDNA from 1 µg of total RNA. qPCR reactions were carried out with SYBR Green qPCR Master Mix (AG11701, Accurate Biology) on a LightCycler 480 II real-time PCR system (Roche). Cycling conditions and primer efficiencies were validated prior to experiments. Gene expression was normalized to GAPDH and relative transcript levels were calculated using the 2⁻ΔΔCt method. All reactions were run in technical triplicates. Primer sequences used in this study are listed in Table 1.

**Table 1:** Primer sequences for quantitative real-time PCR

Target (GenBank accession no.)	Primers
c-Fos (NM_010234.3)	F: ACAGCCTTTCCTACTACCATTCC
	R: GGCACTAGAGACGGACAGATC
CTSK (NM_007802.4)	F: AGCAGAACGGAGGCATTGAC
	R: ATTTAGCTGCCTTTGCCGTG
DC-STAMP (NM_029422.4)	F: TTCTCGTGTCAGTCTCCTTCTACC
	R: TTTCCCGTCAGCCTCTCTCAA
OC-STAMP (NM_029021.1)	F: CCACTGTCCCAATCACACTCA
	R: GTGGTAGATGACAGTCGTGGG
NFATc1 (NM_001164109.1)	F: AGTCTCACCACAGGGCTCAC
	R: TCAGCCGTCCCAATGAACAG
MMP9 (NM_013599.5)	F: GCCCTGGAACTCACACGACA
	R: TTGGAAACTCACACGCCAGAAG
GAPDH (XM_036165840.1)	F: TGTGTCCGTCGTGGATCTGA
	R: TTGCTGTTGAAGTCGCAGGAG
TNF-α (NM_013693.3)	F: ACTCCAGGCGGTGCCTATGT
	R: GTGAGGGTCTGGGCCATAGAA
IL-1β (NM_008361.4)	F: TCCAGGATGAGGACATGAGCAC
	R: AACGTCACACACCAGCAGGTTA
IL-6 (NM_031168.2)	F: TGATGGATGCTACCAAACTGGA
	R: TCTCTCTGAAGGACTCTGGCT

*c-Fos* Fos proto-oncogene, AP-1 transcription factor subunit, *CTSK* cathepsin K, *DC-STAMP* dendritic cell–specific transmembrane protein, *OC-STAMP* osteoclast stimulatory transmembrane protein, NFATc1, nuclear factor of activated T-cells 1, *MMP9* matrix metallopeptidase 9, *GAPDH* glyceraldehyde-3-phosphate dehydrogenase, *TNF-α* tumor necrosis factor alpha, *IL-1β* interleukin-1 beta, *IL-6* interleukin-6, *F* Forward Primer, *R* Reverse Primer

### Western Blot

Western blotting was performed as previously described [19]. Cells were lysed with pre-chilled RIPA buffer (R0020, Solarbio) containing 1% protease (CW2200, Cwbio) and phosphatase inhibitors (CW2383, Cwbio). Protein levels were quantified using a BCA kit (PC0020, Solarbio). Equal protein samples (20–30 µg) were denatured at 95 °C, separated by 10% SDS-PAGE, and transferred onto 0.2 μm PVDF membranes (Millipore). After blocking with 5% non-fat milk for 30–60 min, membranes were incubated overnight at 4 °C with primary antibodies, followed by HRP-conjugated secondary antibodies (Proteintech) for 1–2 h at room temperature. Protein bands were detected using ECL substrate (Bio-Rad) and analyzed with ImageJ software.

### Enzyme-linked Immunosorbent Assays

Enzyme-linked immunosorbent assays (ELISAs) were performed using Mouse TNF-α (Proteintech, KE10002), IL-1β (Proteintech, KE10003), and IL-6 ELISA Kit (Proteintech, KE10007) according to the manufacturers’ instructions. All reagents were equilibrated to room temperature for 20–30 min before use. The required microplate strips were taken out, and 100 µL of standards or samples were added to each well, followed by incubation at 37 °C for 2 h. After washing the plate four times, 100 µL of 1× detection antibody was added to each well and incubated at 37 °C for 1 h. The plate was washed four times again, and 100 µL of 1× HRP-conjugated streptavidin was added, followed by incubation at 37 °C for 40 min. Following another four washes, 100 µL of TMB substrate solution was added to each well and incubated at 37 °C in the dark for 15–20 min. The reaction was terminated by adding 100 µL of stop solution to each well, and absorbance was immediately measured at 450 nm with wavelength correction at 630 nm. Cytokine concentrations were calculated based on standard curves.

### Bone Resorption Assay

Bone resorption assays were performed using hydroxyapatite-coated plates (Plate S96, Lot No. 23C890, Cosmo Bio). Briefly, plates were pretreated with FACS labeling solution under sterile conditions and incubated in the dark for 1–2 h, followed by two gentle washes with sterile PBS and preconditioning with α-MEM for 12–24 h. Cells were then seeded onto the plates and differentiated into osteoclasts according to standard protocols. To evaluate hydroxyapatite resorptive activity, cells were additionally stimulated with M-CSF and RANKL or LPS at the same concentrations for an additional 2 days starting on day 5 of differentiation. On day 7, fluorescence images were first acquired using a fluorescence microscope, after which the culture medium was removed and cells were eliminated by treatment with 5% sodium hypochlorite for 3–5 min. Plates were subsequently washed three times with ddH₂O and air-dried at room temperature. Resorption pits were visualized by light microscopy and quantified using ImageJ software, with resorbed area expressed as a percentage of the total surface area.

### Establishment of Mouse Calvarial Osteolysis Model

The inflammatory calvarial osteolysis model has been widely validated in female mice, which exhibit more stable and pronounced osteolytic phenotypes, thereby enhancing experimental reproducibility. Additional, their relatively stable estrogen levels reduce inter-individual variability in bone turnover, allowing for clearer evaluation of treatment-specific effects by minimizing hormonal background noise [38–41]. We therefore assessed the protective effect of 6-HOF against inflammatory bone loss on female mice using a slightly modified LPS-induced calvarial osteolysis model.

Fifteen female C57BL/6J mice (6–8 weeks old, 26 ± 2 g) were randomly assigned to three groups (*n* = 5 per group): sham-operated, LPS model, and LPS + 6-HOF treatment. Mice in the LPS model and treatment groups received subcutaneous injections of LPS (5 mg/kg) over the sagittal suture on days 1, 3, 5, 7, 9, 11, and 13, while the sham group received equivalent volumes of saline. The treatment group additionally received 6-HOF (10 mg/kg) on days 2, 4, 6, 8, 10, 12, and 14, whereas the other groups received PBS at the same time points. All procedures were performed under anesthesia. Animals were maintained under specific-pathogen-free (SPF) conditions with controlled temperature (22 °C) and a 12-h light/dark cycle; no mortality or abnormal behaviors were observed. Mice were sacrificed on day 15, and calvariae were harvested and fixed in 4% paraformaldehyde (PFA) for micro-CT analysis.

### Micro-CT Analysis of Bone Tissue

After fixation in 4% paraformaldehyde for 24 h, calvarial samples were transferred to 70% ethanol for gradient dehydration. High-resolution micro-computed tomography (micro-CT) scanning was performed using a vivaCT 80 system (SCANCO) with a spatial resolution of 14 μm, tube voltage of 70 kVp, and current of 114 µA. Three-dimensional bone structures were reconstructed using Mimics Research 21.0 software. A standardized square region of interest (ROI) surrounding the midline suture of the calvaria was defined for quantitative analysis. Bone resorption was evaluated by calculating bone volume fraction (BV/TV) and cortical porosity (Ct.Po) parameters.

### Statistical Analysis

All in vitro data were obtained from three independent experiments performed using distinct biological samples, while in vivo analyses were conducted with five independent biological replicates. The sample size for each statistical analysis corresponds to the number of biological replicates. Quantitative data are presented as the mean ± standard deviation (SD). Statistical significance between two groups was evaluated using an unpaired Student’s *t*-test, whereas comparisons among multiple groups were assessed by one-way analysis of variance (ANOVA). A *p* value < 0.05 was considered statistically significant. All statistical analyses were performed using GraphPad Prism software (Version 10,GraphPad Software, San Diego, CA, USA).

## **Result**

### Potential Targets and Signaling Pathways of 6-HOF

**Fig. 1:**
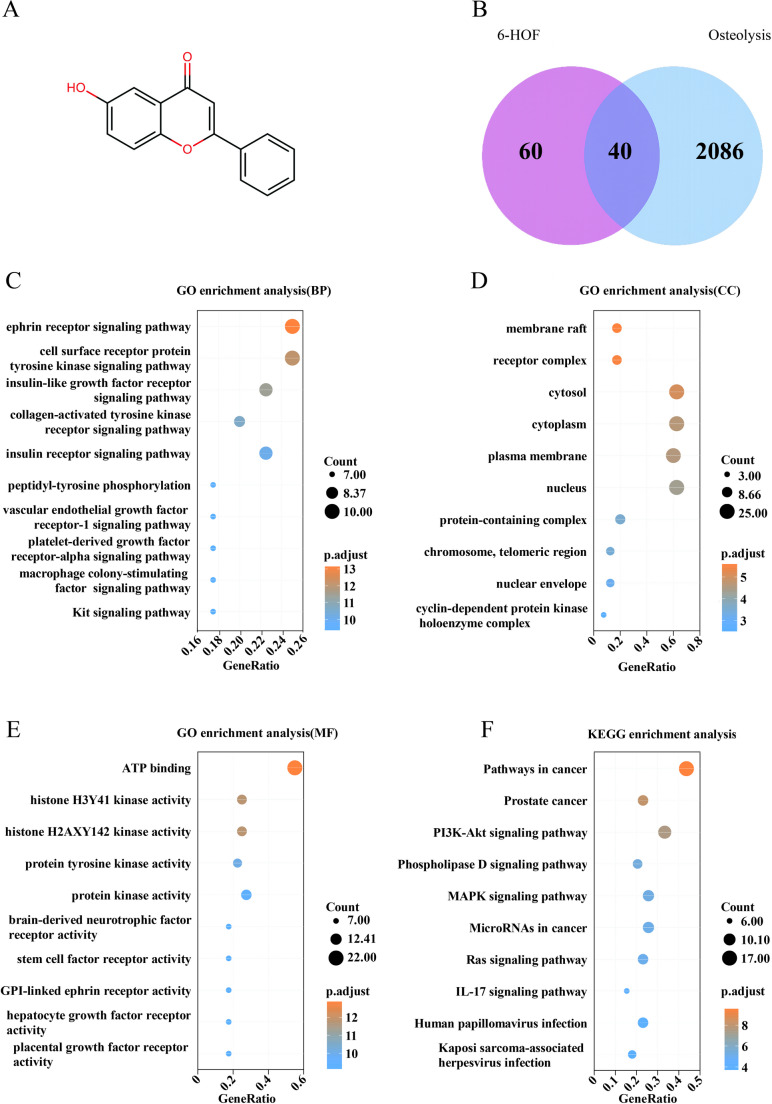
Potential targets of 6-HOF in the prevention of inflammatory osteolysis predicted by GO/KEGG enrichment. **A** Molecular structure of 6-HOF. **B** The Venn diagram shows the shared targets between 6-HOF and inflammatory osteolysis. **C**–**E** GO enrichment of the 40 overlapping target genes, showing the top 10 terms in biological process (BP), molecular function (MF), and cellular component (CC). **F** KEGG pathway enrichment of the 40 target genes, highlighting the top 10 associated signaling pathways

**Fig. 2:**
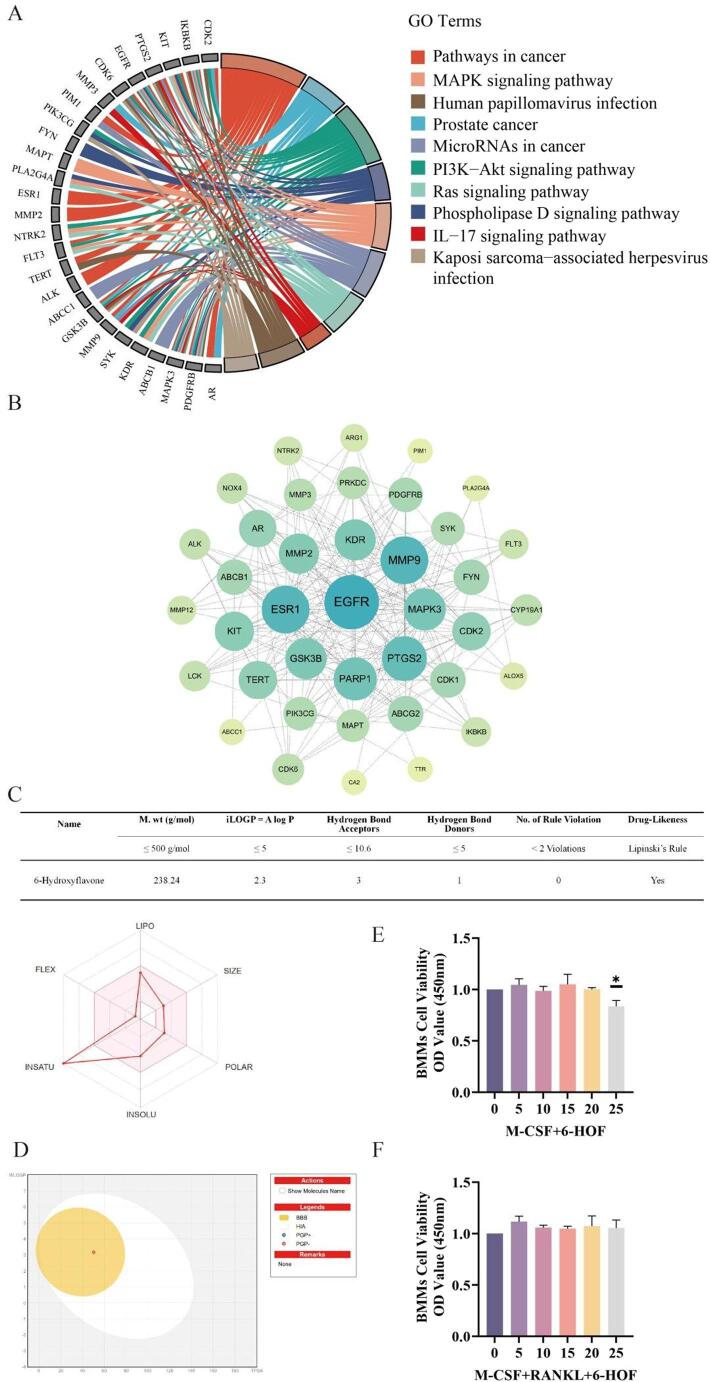
Comprehensive analysis of potential targets, pharmacokinetic profiles, and cytotoxicity of 6-HOF. **A** Pathway enrichment analysis was performed based on the shared targets between inflammatory osteolysis and 6-HOF, and the associations between genes and pathways were visualized using a chord diagram. **B** The overall protein–protein interaction (PPI) network was established, revealing the interaction landscape of 39 potential target proteins. **C** Radar plot analysis quantified six molecular descriptors: SIZE (molecular weight:238.24 g/mol), LIPO (XLOGP3:3.62), POLAR (TPSA: 50.44 Å²), INSOLU (Log S (ESOL):−4.19), INSATU (fraction Csp³:0), and FLEX (number of rotatable bonds:1). The pink zone represents the optimal range for oral bioavailability. **D** The BOILED-Egg model predicted by SwissADME (WLOGP = 3.17, TPSA = 50.44 Å²) located 6-HOF in both the yolk (BBB-permeable) and white (GI-absorbable) regions, indicating its dual absorption potential. **E** The cytotoxicity of 6-HOF on bone marrow–derived macrophages (BMMs) was evaluated using the CCK-8 assay. BMMs were treated with M-CSF (10 ng/mL) and various concentrations of 6-HOF for 24 h. Data are presented as mean ± SD (*n* = 3). *P < 0.05. **F** BMMs were further cultured with M-CSF (10 ng/mL) and RANKL (30 ng/mL) in the presence of different concentrations of 6-HOF, under conditions identical to those in (**E**). Results are shown as mean ± SD (*n* = 3); ns, not significant

To investigate the potential molecular mechanisms by which 6-HOF alleviates inflammatory osteolysis, network pharmacology analysis was performed. The 2D chemical structure of 6-HOF was input into the Swiss Target Prediction platform, yielding 100 predicted drug-target genes (Fig. 1A). The full list of predicted targets for 6-Hydroxyflavone is presented in Supplementary Material 2. Meanwhile, using “Osteolysis” as a keyword, a total of 2126 osteolysis-related human genes were retrieved from the GeneCards and OMIM databases. Intersection analysis identified 40 common targets potentially associated with both 6-HOF and osteolysis (Fig. 1B; Table 2). The corresponding detailed results are provided in Supplementary Materials 3–5. These 40 overlapping targets were subjected to GO and (KEGG enrichment analyses using DAVID (*p* < 0.05, count ≥ 2), and the top 10 enriched biological processes (BP), cellular components (CC), molecular functions (MF), and KEGG pathways were extracted. The results indicated that these targets were mainly involved in osteoclast differentiation and regulation of oxidative stress, particularly enriched in receptor tyrosine kinase signaling localized to the plasma membrane and lipid rafts, such as M-CSF and PDGF receptor-mediated pathways (Fig. 1C–F). Detailed information is provided in Supplementary Material 6. Further KEGG-based gene–pathway circos analysis revealed that these targets were also associated with PI3K–Akt, MAPK, Ras, IL-17, phospholipase D signaling (Fig. 2A). These pathways are known to play critical roles in osteoclast differentiation, amplification of inflammatory signals, and regulation of bone resorption [42, 43]. Moreover, a protein–protein interaction (PPI) network was constructed using the STRING database, comprising 39 potential target proteins and their interactions (Fig. 2B). Network topology analysis identified EGFR, ESR1, MMP9, PTGS2, MAPK3, KDR, and PARP1 as high-degree nodes in the network core, suggesting that these hub targets may play pivotal roles in mediating the effects of 6-HOF on inflammatory osteolysis. Taken together, these findings suggest that 6-HOF may exert anti-osteolytic effects by modulating osteoclast differentiation, oxidative stress, and inflammatory responses through a multi-target, multi-pathway network.

**Table 2:** Overlapping target genes 6-HOF and osteolysis

MMP2	CDK1	CYP19A1	PDGFRB	FYN	KIT	PTGS2	PRKDC	ESR1	GSK3B
MMP12	MMP3	NTRK2	EGFR	IKBKB	TERT	CDK2	ABCC1	ALOX5	ALK
MAPT	ABCB1	PIM1	CDK6	LCK	PLA2G4A	ARG1	NOX4	PLAA	PARP1
KDR	ABCG2	TTR	PIK3CG	FLT3	SYK	MAPK3	CA2	AR	MMP9

### Physicochemical and Pharmacokinetic Profiling of 6-HOF

The pharmacokinetic and physicochemical properties of 6-HOF were predicted using Lipinski’s Rule of Five and the SwissADME platform. All molecular descriptors—including molecular weight, lipophilicity, polarity, and hydrogen-bond donors/acceptors—conformed to Lipinski’s criteria, indicating favorable drug-likeness (Fig. 2C).

The bioavailability radar plot demonstrated that 6-HOF parameters, including size, polarity, solubility, and flexibility, all fall within the optimal range for oral absorption. The BOILED-Egg model predicted efficient gastrointestinal absorption and blood–brain barrier (BBB) penetration (Fig. 2D). Positioned within the overlapping GI and BBB zones, 6-HOF exhibited a WLOGP of 3.17 and TPSA of 50.44 Å² and was identified as a non-P-glycoprotein substrate, implying minimal efflux potential. Together, these data indicate that 6-HOF possesses ideal pharmacokinetic characteristics supporting its potential as an orally bioavailable natural compound.

### 6-HOF Suppresses Inflammatory Osteoclastogenesis during the Differentiation Stage without Affecting BMM Viability In Vitro

To assess the cytotoxic effects of 6-HOF and determine a safe working concentration, cell viability was first evaluated using a CCK-8 assay in bone marrow–derived monocytes/macrophages (BMMs). BMMs were cultured with M-CSF alone or in combination with RANKL, and then treated with different concentrations of 6-HOF for 24 h. No significant changes in cell viability were observed at concentrations up to 20 µM, whereas a slight reduction was noted at 25 µM **(**Fig. 2E–F**)**. Therefore, 20 µM was selected as the maximal working concentration for all subsequent in vitro experiments.

**Fig. 3:**
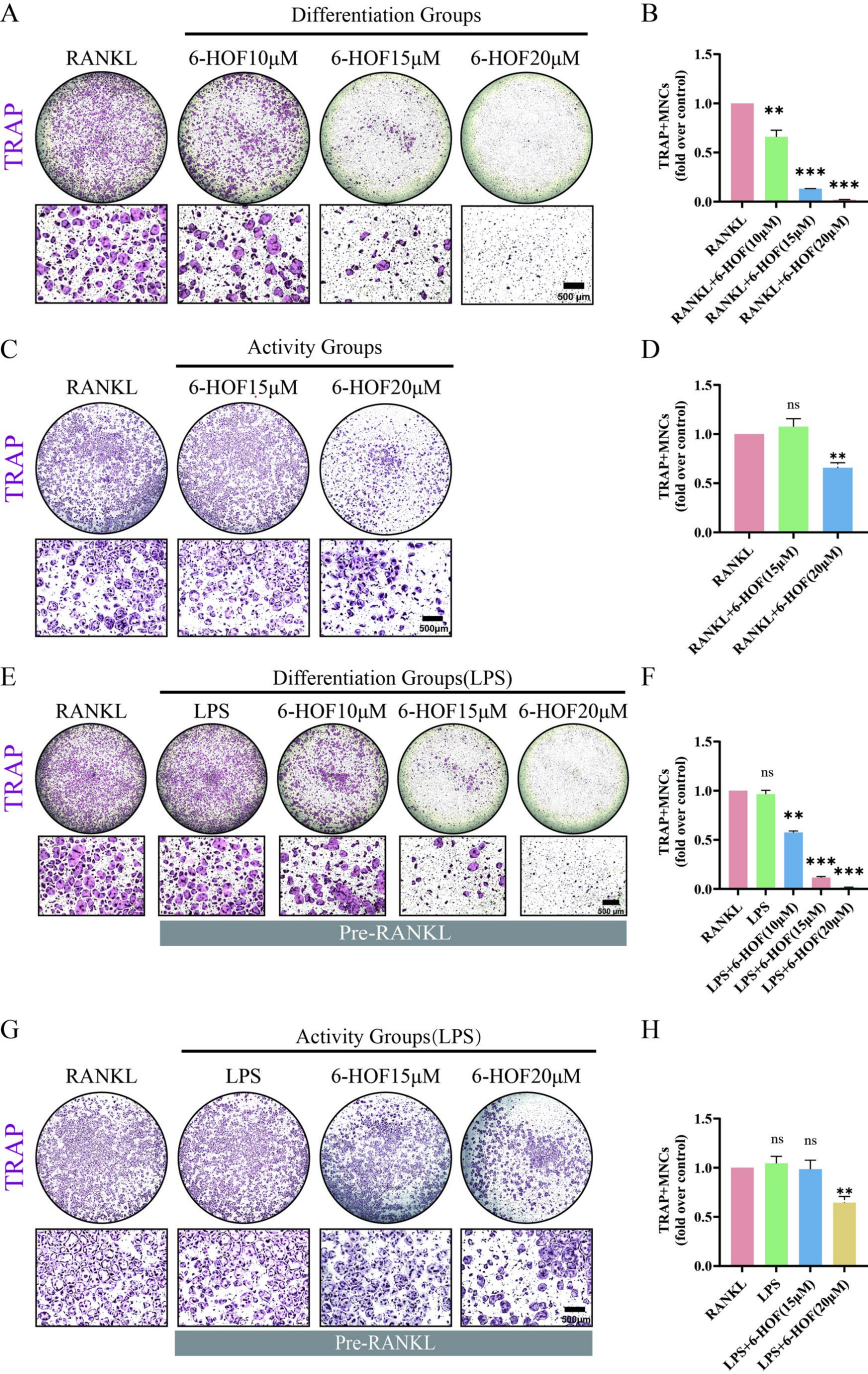
6-HOF suppresses inflammatory osteoclastogenesis during the differentiation stage in vitro. **A** TRAP staining of osteoclasts differentiated from BMMs in the differentiation group treated with different concentrations of 6-HOF. Scale bar = 500 μm. **B** Quantification of TRAP-positive multinucleated osteoclasts in (**A**). Data are presented as mean ± SD (*n* = 3). **p < 0.01, ***p < 0.001; ns, not significant. **C** TRAP staining of mature osteoclasts in the activity group treated with different concentrations of 6-HOF. Scale bar = 500 μm. **D** Quantification of TRAP-positive multinucleated osteoclasts in (**C**), analyzed as described in (**B**) (*n* = 3). **E** TRAP staining showing the effects of different treatment schedules of 6-HOF on LPS-induced osteoclastogenesis in the differentiation group. Scale bar = 500 μm. **F** Quantification of TRAP-positive multinucleated osteoclasts in (**E**), analyzed as described in (**B**) (*n* = 3). **G** TRAP staining showing the effects of different treatment schedules of 6-HOF on LPS-induced mature osteoclast activity in the activity group. Scale bar = 500 μm. **H** Quantification of TRAP-positive multinucleated osteoclasts in (**G**), analyzed as described in (**B**) (*n* = 3)

Based on the CCK-8 results, TRAP staining was performed to assess the effect of 6-HOF on osteoclast formation under various conditions. Under RANKL-induced conditions, 6-HOF treatment significantly reduced the formation of TRAP-positive multinucleated osteoclasts in a concentration-dependent manner, with the strongest inhibitory effect observed at 20 µM (Fig. 3A–B). An in vitro inflammatory osteoclastogenesis model was then established as previously described [36, 37]. The results showed that LPS treatment promoted osteoclast formation in a dose-dependent manner, with 200 ng/mL LPS inducing osteoclastogenesis to a level comparable to that observed with RANKL stimulation alone (Fig. 3E–F). Accordingly, 200 ng/mL LPS was selected for use in subsequent experiments.

In the LPS-induced inflammatory model, 6-HOF significantly reduced the number of TRAP-positive multinucleated osteoclasts in the differentiation group, exhibiting an inhibitory trend comparable to that observed under RANKL stimulation (Fig. 3E–F). These results indicate that 6-HOF suppresses inflammatory osteoclastogenesis without affecting BMM viability. 

To further investigate whether 6-HOF affects osteoclasts after the initiation of inflammatory stimulation, an activity-stage treatment protocol was performed as described in Sect. Cell culture and osteoclast differentiation. Compared with the corresponding control groups, treatment with 15 µM 6-HOF during the activity stage did not significantly reduce the number of TRAP-positive multinucleated osteoclasts; however, the 20 µM group exhibited a marked inhibitory effect, reaching statistical significance (Fig. 3C–D, G–H).

Collectively, these results indicate that 6-HOF exerts more robust and consistent inhibitory effects during the differentiation stage, effectively restraining osteoclast formation. Notably, even after the differentiation program has been initiated, 6-HOF retains the capacity to interfere with osteoclast formation.

### 6-HOF Disrupts Osteoclast Cytoskeletal Organization and Bone-Resorptive Function

**Fig. 4:**
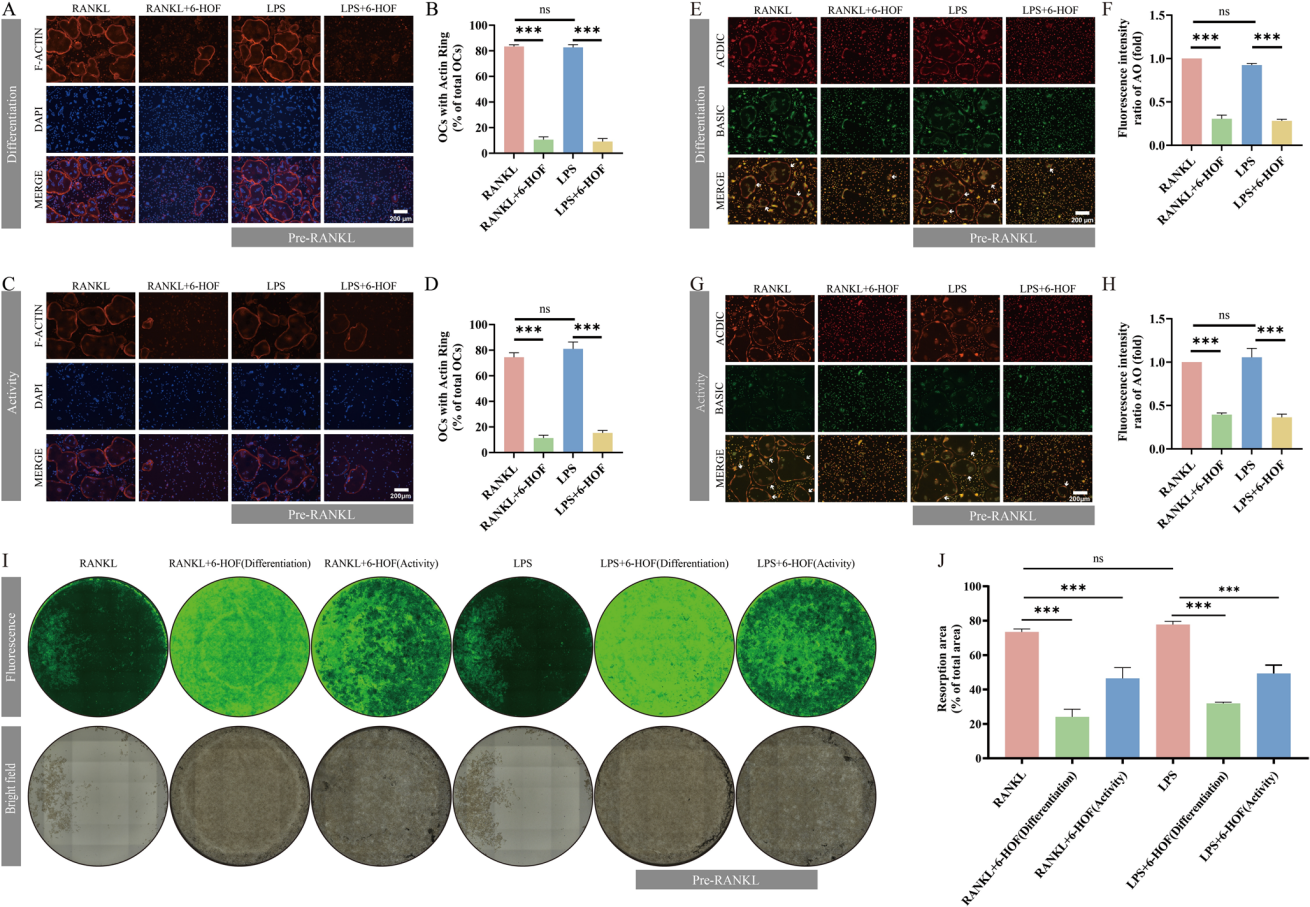
6-HOF Disrupts Osteoclast Cytoskeletal Organization and Bone-Resorptive Function. **A** Representative confocal images of F-actin ring formation in osteoclasts from the RANKL, LPS, LPS + 6-HOF, and RANKL + 6-HOF groups in the differentiation group, stained with phalloidin and DAPI. Scale bar, 200 μm. **B** Quantification of F-actin ring–positive osteoclasts expressed as a percentage of total osteoclasts. **C** Representative confocal images of F-actin rings in osteoclasts from the RANKL, LPS, LPS + 6-HOF, and RANKL + 6-HOF groups in the activity group. Scale bar, 200 μm. **D** Quantification of F-actin ring–positive osteoclasts in (**C**). **E** Representative confocal images of acidic vesicles in osteoclasts from the RANKL, LPS, LPS + 6-HOF, and RANKL + 6-HOF groups in the differentiation group, detected by acridine orange (AO) staining. White arrows indicate acidic vesicles. Scale bar, 200 μm. **F** Quantification of AO fluorescence intensity in (**E**). **G** Representative confocal images of AO-stained acidic vesicles in osteoclasts from the RANKL, LPS, LPS + 6-HOF, and RANKL + 6-HOF groups in the activity group. Scale bar, 200 μm. **H** Quantification of AO fluorescence intensity in (**G**). **I** Representative images of bone resorption pits. Upper panels show fluorescence images, and lower panels show corresponding bright-field images of the same fields. Osteoclasts were treated with 20 μM 6-HOF. **J** Quantification of bone resorption area. Data are presented as mean ± SD (*n* = 3). ns, not significant; ***P < 0.001

F-actin ring assembly and intracellular acidification, two key functional prerequisites for osteoclast activity (bone resorption), were examined to evaluate the effects of 6-HOF on osteoclast activity. Phalloidin-iFluor 594 and AO staining were employed to visualize cytoskeletal organization and acidic vesicle formation, respectively. Under RANKL or LPS stimulation, most mature osteoclasts displayed well-organized F-actin rings, with 83.41% of cells in the RANKL group and 82.71% in the LPS group exhibiting intact actin structures. In contrast, exposure to 6-HOF resulted in a pronounced disruption of actin ring formation, reducing the proportion of osteoclasts with complete F-actin rings to 10.61% in the RANKL + 6-HOF group and 9.14% in the LPS + 6-HOF group (Fig. 4A–B). To further determine whether 6-HOF affects osteoclast activity after the onset of inflammatory stimulation, an activity-treatment protocol was applied as described in the Methods Sect. Cell culture and osteoclast differentiation. Compared with the corresponding control groups, osteoclasts in the 6-HOF (activity) groups exhibited a markedly lower frequency of intact F-actin ring formation (Fig. 4C–D).

Consistent with these structural alterations, AO staining revealed abundant acidic vesicles in osteoclasts stimulated with RANKL or LPS, whereas 6-HOF treatment markedly diminished the red/green fluorescence ratio in both differentiation (Fig. 4E–F) and activity (Fig. 4G–H) groups, indicating a severe impairment of acidification capacity. These findings suggest that 6-HOF compromises osteoclast functionality by disrupting both cytoskeletal integrity and proton secretion.

Functional consequences of these alterations were further validated using a bone resorption pit assay. Extensive and continuous resorption lacunae were observed on biomimetic substrates under RANKL or LPS stimulation, whereas both differentiation-treatment and activity-treatment of 6-HOF markedly reduced the number and area of resorption pits (Fig. 4I–J). Quantitative analysis confirmed a significant decrease in total resorbed surface area in all 6-HOF–treated groups relative to their respective controls. Taken together, these results demonstrate that 6-HOF not only suppresses osteoclast differentiation but also persistently disrupts cytoskeletal organization, acidification capacity, and bone-resorptive function even after the differentiation process is nearly completed, indicating a sustained inhibitory effect on osteoclast activity under inflammatory conditions.

### 6-HOF Attenuates LPS-induced Osteoclastogenesis-related Gene Expression and Inflammatory Cytokine Production

**Fig. 5:**
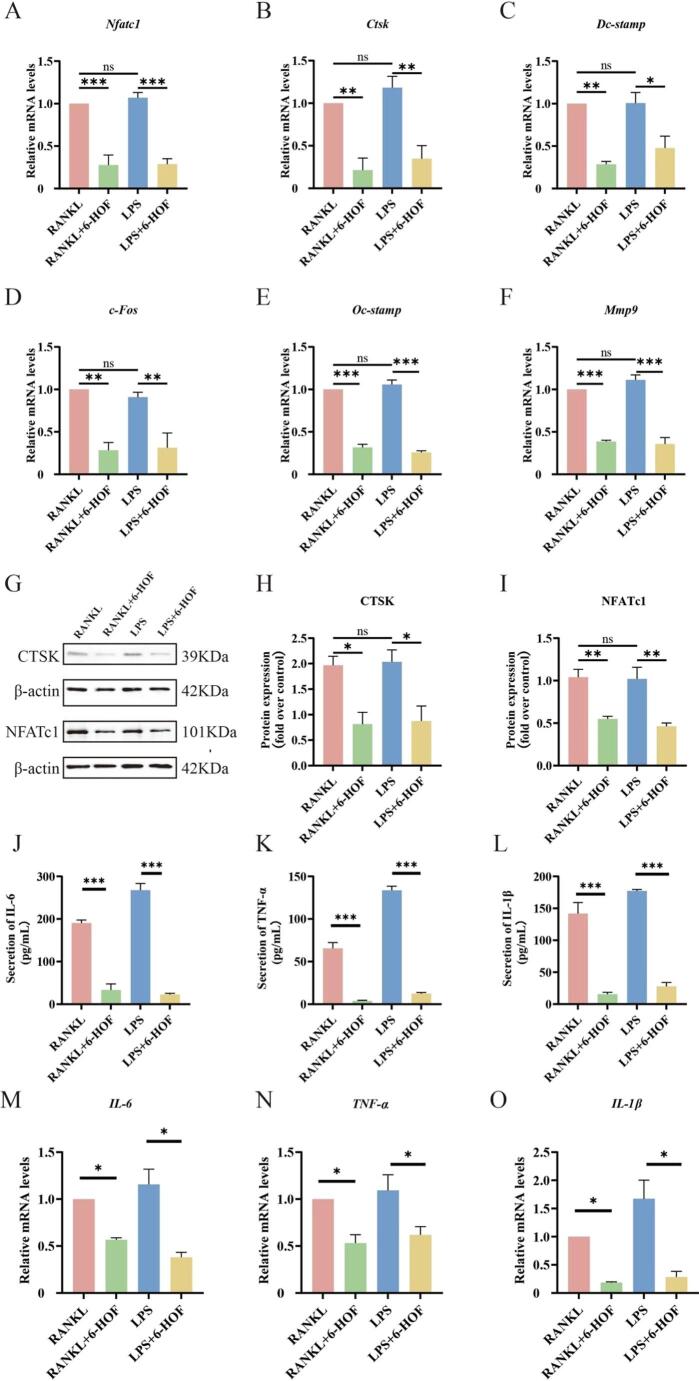
6-HOF attenuates LPS-induced osteoclastogenesis-related gene expression and inflammatory cytokine production. **A**–**F** RT–qPCR analysis of the mRNA expression levels of Nfatc1, Ctsk, Dc-stamp, c-Fos, Oc-stamp, and Mmp9 in osteoclasts treated with or without 6-HOF. GAPDH was used as an internal control. Data are presented as mean ± SD (*n* = 3). ns, not significant; *P < 0.05, **P < 0.01, ***P < 0.001. **G** Representative Western blot images showing the protein expression of CTSK and NFATc1 in osteoclasts from different experimental groups. **H**–**I** Densitometric quantification of Western blot bands using ImageJ software, normalized to β-actin. Data are presented as mean ± SD (*n* = 3). ns, not significant; *P < 0.05, **P < 0.01. **J**–**L** Enzyme-linked immunosorbent assay (ELISA) analysis of IL-6, TNF-α, and IL-1β levels in the culture supernatants of osteoclasts from different treatment groups. Data are presented as mean ± SD (*n* = 3). *P < 0.05. **M**–**O** RT–qPCR analysis of the mRNA expression levels of Il-6, Tnf-α, and Il-1β in osteoclasts treated with or without 6-HOF. Data are presented as mean ± SD (*n* = 3). *P < 0.05

The molecular mechanisms underlying the inhibitory effects of 6-HOF on osteoclastogenesis were examined under LPS stimulation. During the differentiation stage, qRT–PCR analysis demonstrated that 6-HOF treatment markedly reduced the mRNA levels of the key osteoclastogenic transcription factors Nfatc1 and c-Fos, as well as osteoclast functional genes including Ctsk, Mmp9, Dc-stamp, and Oc-stamp (Fig. 5A–F). Consistently, Western blot analysis further confirmed that the addition of 6-HOF during the differentiation stage significantly decreased NFATc1 and CTSK protein expression (Fig. 5G–I), indicating suppression of osteoclast differentiation and functional maturation at both transcriptional and translational levels.

Given the critical role of inflammatory cytokines in LPS-mediated osteoclast activation, the effects of 6-HOF on inflammatory mediator production during the activity stage were subsequently evaluated. qRT–PCR results showed that LPS markedly upregulated the transcription of Il-6, Tnf-α, and Il-1β in BMMs, whereas the addition of 6-HOF during the activity stage significantly attenuated the expression of these pro-inflammatory genes (Fig. 5M–O). In line with these findings, ELISA assays demonstrated that treatment with 6-HOF during the activity stage substantially reduced the secretion levels of IL-6, TNF-α, and IL-1β in the culture supernatants (Fig. 5J–L).

Collectively, these data indicate that under inflammatory conditions, 6-HOF suppresses osteoclast-related gene expression and inflammatory cytokine production, thereby interfering with LPS-induced osteoclast differentiation and activation.

### 6-HOF Suppresses LPS-induced Reactive Oxygen Species Production and Calcium Oscillations in Osteoclast Precursors

**Fig. 6:**
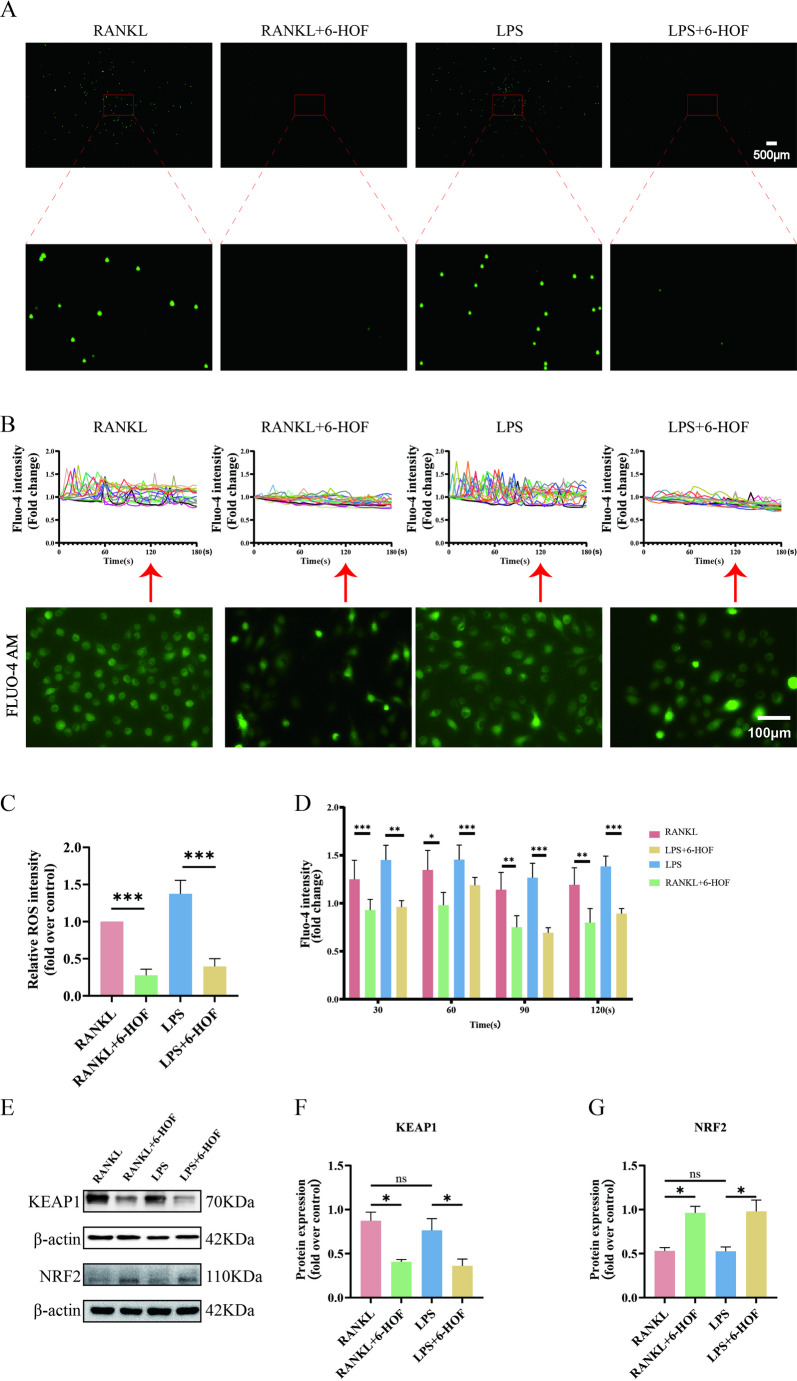
6-HOF suppresses LPS-induced reactive oxygen species production and calcium oscillations in osteoclast precursors. **A** Representative fluorescence images showing intracellular reactive oxygen species (ROS) in osteoclast precursors stained with DCFH-DA. Scale bar = 500 μm. **B** Osteoclast precursors were loaded with Fluo-4 AM to monitor intracellular Ca²⁺ dynamics, and calcium oscillations were recorded using confocal fluorescence microscopy. Twenty cells were randomly analyzed per group, and fluorescence intensity was normalized to the initial value to quantify the amplitude of calcium oscillations. Line graphs depict the dynamic changes in calcium oscillations. Red arrows indicate the Fluo-4 AM fluorescence intensity at 5 s in each group. Scale bar = 200 μm. **C** Quantification of overall fluorescence intensity in control and treated groups using ImageJ software to assess intracellular ROS levels. Data are presented as mean ± SD (*n* = 3). ***P < 0.001. **D** Statistical analysis of Fluo-4 AM fluorescence intensity at specific time points (30 s, 60 s, 90 s, and 120 s). Data are presented as mean ± SD (*n* = 3). *P < 0.05, **P < 0.01, ***P < 0.001. **E**–**G** Western blot analysis of Nrf2 and Keap1 protein expression in osteoclasts, with β-actin used as a loading control for densitometric quantification. Data are presented as mean ± SD (*n* = 3). ns, not significant; *P < 0.05

Accumulating evidence indicates that excessive ROS production enhances osteoclast activity and accelerates inflammatory osteolysis, while calcium oscillations constitute a critical upstream signaling event during RANKL-induced osteoclast differentiation [44–46]. Consistent with these observations, our bioinformatic analyses identified ROS metabolism and calcium signaling as key pathways associated with 6-HOF intervention (Fig. 1C–F). DCFH-DA fluorescence staining demonstrated that 6-HOF markedly attenuated both the rapid ROS burst induced by LPS and the sustained ROS generation triggered by RANKL stimulation (Fig. 6A, C). In parallel, calcium imaging analyses revealed a pronounced reduction in the frequency and amplitude of intracellular calcium oscillations in osteoclast precursors following 6-HOF treatment (Fig. 6B). Quantitative analysis of Fluo-4 AM fluorescence intensity at 30 s, 60 s, 90 s, and 120 s further confirmed that 6-HOF effectively suppressed calcium oscillatory activity during osteoclastogenesis (Fig. 6D). Moreover, Western blot analysis showed decreased Keap1 expression accompanied by a significant upregulation of Nrf2, indicating activation of the Nrf2-dependent antioxidant defense system (Fig. 6E–G). Collectively, these findings demonstrate that 6-HOF inhibits osteoclastogenesis through coordinated regulation of oxidative stress and calcium signaling pathways.

### 6-HOF Mitigates LPS-Induced Calvarial Osteolysis In Vivo

**Fig. 7:**
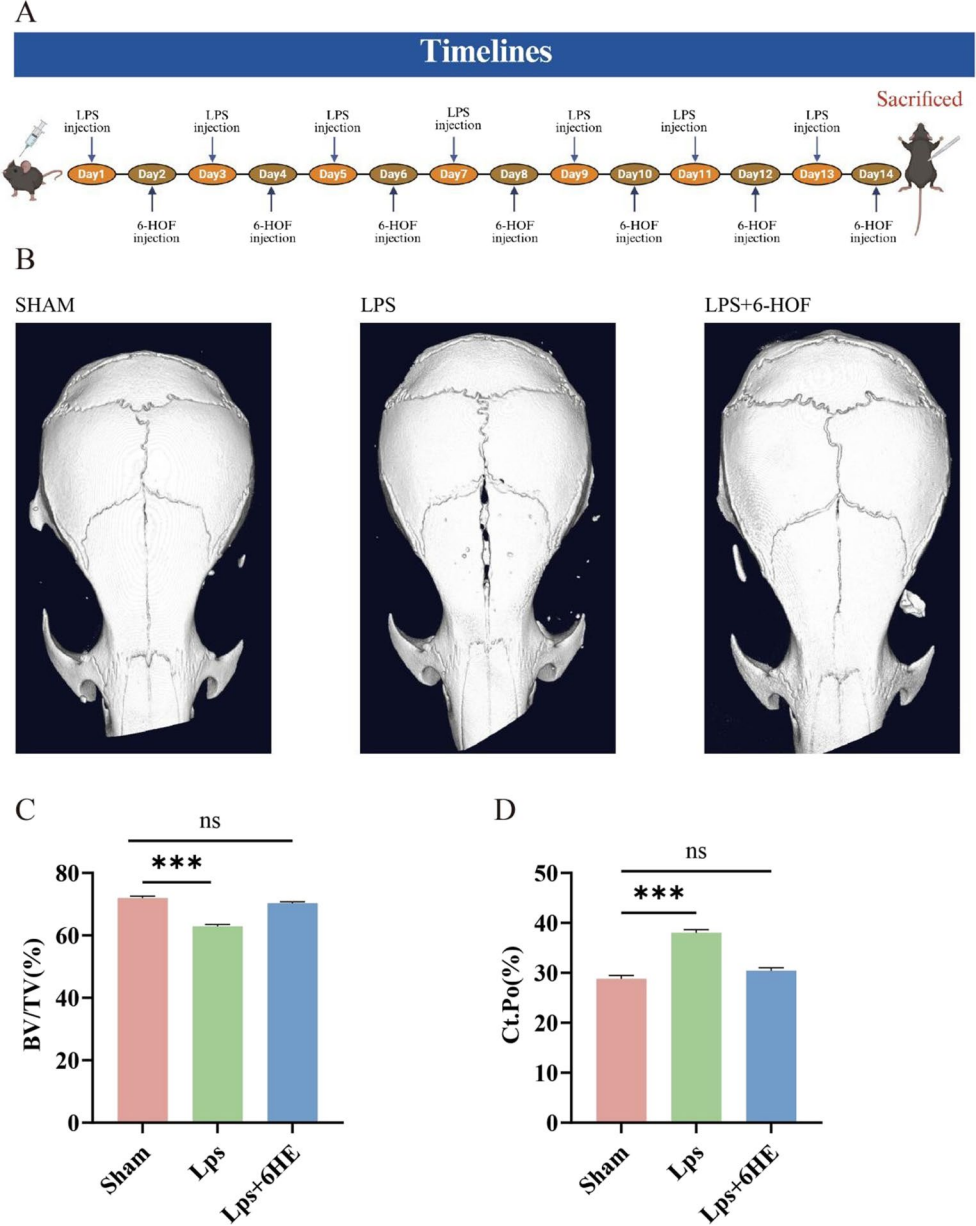
6-HOF alleviates LPS-induced cranial bone resorption. **A** Schematic timeline of the animal experiment. **B** Representative 3D micro-CT reconstruction images of mouse calvaria from each group. **C**-**D** Quantitative analysis of bone volume fraction (BV/TV) and cortical bone porosity. Data are shown as mean ± SD, ***p < 0.001

An LPS-induced calvarial osteolysis model was used to validate the in vivo efficacy of 6-HOF (Fig. 7A). Micro-CT reconstruction revealed extensive bone erosion in the LPS group, which was significantly reversed following 6-HOF treatment (Fig. 7B). Quantitative morphometric analysis showed that 6-HOF restored BV/TV and reduced cortical porosity compared with LPS controls (Fig. 7C–D). These findings demonstrate that 6-HOF effectively alleviates LPS-induced inflammatory osteolysis in vivo by preventing excessive bone resorption.

## Discussion

**Fig. 8:**
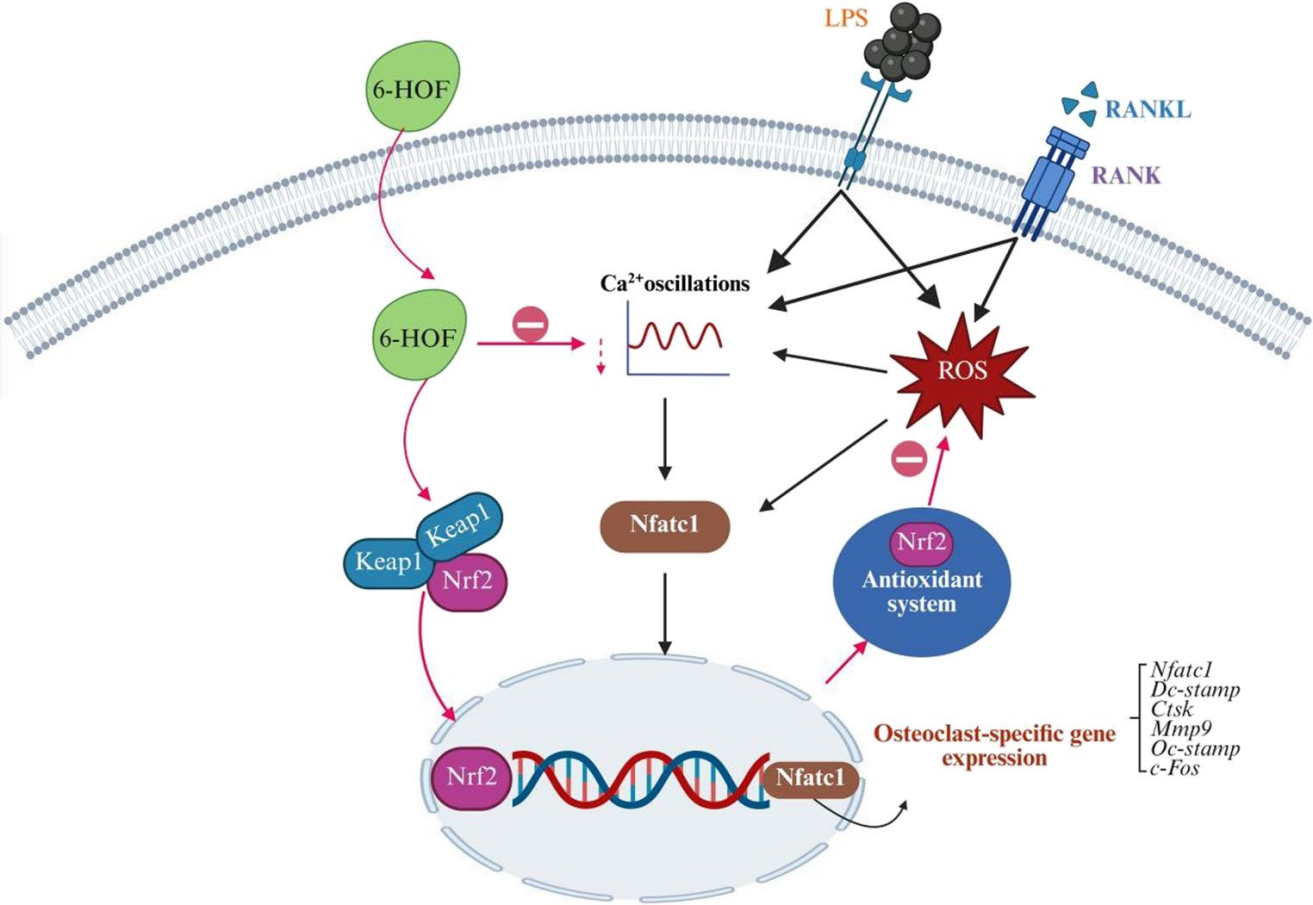
Proposed mechanism by which 6-HOF inhibits LPS-induced osteoclastogenesis. LPS stimulation induces oxidative stress and Ca²⁺ oscillations, promoting osteoclast proliferation and activation. 6-HOF activates the Keap1/Nrf2 antioxidant pathway to scavenge excessive ROS, thereby suppressing Nfatc1 activation. In parallel, inhibition of Ca²⁺ oscillations further limits Nfatc1 activity, synergistically restraining osteoclast formation

Inflammatory osteolysis is a bone-destructive disorder driven by immune dysregulation and an imbalanced inflammatory microenvironment, characterized by excessive osteoclast activation that accelerates bone resorption beyond bone formation, leading to bone loss and structural damage [2]. Proinflammatory mediators such as TNF-α, IL-1β, IL-6, and LPS have been shown to continuously activate the RANKL/RANK axis and its downstream NF-κB, MAPK, and NFATc1 pathways, thereby promoting osteoclast differentiation and function and ultimately disrupting bone homeostasis [1, 3]. Clinically, this pathological process commonly occurs in periprosthetic osteolysis, chronic periodontitis, and rheumatoid arthritis, seriously impairing bone regeneration and patient quality of life [5, 6]. Current antiresorptive agents such as bisphosphonates, denosumab, and NSAIDs offer partial benefit but are limited by adverse effects, diminishing efficacy, and post-treatment rebound bone loss [10–12]. In recent years, natural flavonoids have become a key focus in bone metabolism research due to their multitarget regulation and antioxidant effects [20, 21]0.6-HOF, a unique monoflavone, shows strong free radical–scavenging activity and good membrane permeability [20, 23, 24]. This study demonstrates for the first time that 6-HOF activates the cellular antioxidant system, regulates the Keap1/Nrf2 pathway, and suppresses osteoclastic calcium oscillations to restore bone homeostasis and inhibit abnormal osteoclast differentiation (Fig. 8).

This study systematically evaluated the anti-osteolytic potential of 6-HOF through pharmacokinetic analysis, in vitro functional experiments, and in vivo validation. Predictions from the SwissADME platform indicated that6-HOF possesses favorable drug-like properties. Its molecular weight, lipophilicity, polarity, and hydrogen-bonding parameters conform to Lipinski’s rule of five, suggesting good membrane permeability and oral absorption potential [31]. The bioavailability radar revealed that the physicochemical characteristics of 6-HOF fall within the optimal absorption region, while the BOILED-Egg model predicted high gastrointestinal absorption and central nervous system permeability [33]. Furthermore,6-HOF was identified as a non–P-glycoprotein substrate, implying a low tendency for cellular efflux, which may support its pharmacological activity within bone tissues.In the in vitro experiments, CCK-8 assays demonstrated that6-HOF exhibited no significant cytotoxicity toward BMMs at concentrations below 20 µM. Both RANKL and LPS stimulation induced the formation of TRAP-positive multinucleated osteoclasts, whereas 6-HOF treatment markedly reduced osteoclast numbers in a concentration-dependent manner, indicating its potent inhibitory effect on osteoclast differentiation. For osteoclasts, the intact formation of the F-actin ring is essential for attachment to the bone surface and for establishing a sealing zone, while the generation and secretion of acidic vesicles directly determine their bone matrix degradation capacity [18, 35].Further functional assays revealed that 6-HOF significantly disrupted the resorptive structure and acidification function of osteoclasts. Under RANKL and LPS stimulation, approximately 83% of osteoclasts formed intact F-actin rings, whereas this proportion decreased to around 10% after 6-HOF treatment. This reduction was accompanied by a decrease in acidic vesicle formation and a marked attenuation of AO staining fluorescence, suggesting that 6-HOF effectively inhibited osteoclastic acid secretion and bone matrix degradation. At the molecular level, RT-qPCR and Western blot analyses demonstrated that 6-HOF significantly downregulated the expression of NFATc1 and its upstream transcription factor c-Fos in the RANKL–NFATc1 signaling pathway, while simultaneously suppressing multiple downstream effector genes, including Ctsk, Mmp9, Dc-stamp, and Oc-stamp. Given that NFATc1 serves as a master regulator of osteoclast differentiation, these findings indicate that 6-HOF effectively blocks osteoclast formation and bone-resorptive function by inhibiting the RANKL–NFATc1 axis [47].

During the initiation and progression of inflammatory osteolysis, oxidative stress and calcium signaling are widely recognized as two central mechanisms governing osteoclast commitment, functional maturation, and bone-resorptive activity [15, 16]. Increasing evidence suggests that ROS are not merely pathological byproducts of the inflammatory microenvironment but also critical second messengers in osteoclastogenic signaling cascades. While physiological levels of ROS participate in RANKL-induced activation of MAPK and NF-κB pathways, sustained ROS overproduction elicited by inflammatory stimuli such as LPS disrupts mitochondrial homeostasis and establishes positive feedback loops that further amplify osteoclastogenic signaling, thereby markedly accelerating bone resorption [46, 48]. In parallel, RANKL-induced rhythmic Ca²⁺ oscillations represent an indispensable upstream event during osteoclast differentiation, as they activate calcineurin-dependent dephosphorylation and nuclear translocation of the master transcription factor NFATc1, driving continuous transcription of osteoclast-specific genes [44].

Consistent with this established conceptual framework, our GO and KEGG enrichment analyses revealed that the putative targets of 6-HOF are predominantly associated with receptor tyrosine kinase–related signaling pathways localized to the plasma membrane and lipid raft domains, including classical osteoclast regulators such as M-CSF and PDGF, whose downstream effects are coordinated through PI3K–Akt, MAPK, and Ras signaling networks to orchestrate osteoclast differentiation and maturation [42, 49–51]. Importantly, the biological outputs of these pathways are not executed in a linear or isolated manner but critically depend on dynamic regulation of intracellular redox homeostasis and calcium signaling. In this context, ROS and Ca²⁺ oscillations function as key integrative hubs linking receptor-proximal signaling to transcriptional programs: PI3K–Akt and MAPK pathways modulate ROS production through regulation of NADPH oxidase activity, whereas PLCγ, PI3K–Akt, and MAPK signaling cooperatively control calcium influx, endoplasmic reticulum Ca²⁺ release, and calcium homeostasis, thereby finely shaping intracellular Ca²⁺ signaling patterns in osteoclasts [52, 53]. Under inflammatory conditions, stimulation with RANKL or LPS induces sustained ROS accumulation in osteoclast precursors, which in turn amplifies MAPK and NF-κB signaling, and promotes activation and stabilization of NFATc1 [54, 55]. Accordingly, although network enrichment analyses implicate multiple signaling pathways, the pivotal roles of oxidative stress and calcium signaling position them as functional convergence nodes for diverse upstream cues. Guided by this mechanistic convergence, we prioritized oxidative stress and calcium signaling for experimental validation, demonstrating that 6-HOF significantly attenuates LPS- and RANKL-induced ROS overproduction in BMMs. Notably, 6-HOF markedly reduced intracellular ROS fluorescence intensity and restored redox homeostasis, accompanied by downregulation of Keap1 and enhanced activation of Nrf2, indicating the engagement of the Nrf2-mediated antioxidant defense system [56], thereby facilitating clearance of excessive ROS and mitigating oxidative damage, which aligns with our previous findings [35].

In addition to oxidative stress, calcium signaling also serves as a key regulatory pathway in osteoclast differentiation. RANKL stimulation triggers periodic calcium release, leading to oscillatory calcium signals that activate the calcium-dependent phosphatase calcineurin, which in turn promotes NFATc1 dephosphorylation and nuclear transcriptional activation [44, 57]. Calcium imaging results showed that both the frequency and amplitude of intracellular calcium oscillations were markedly increased upon LPS or RANKL stimulation, whereas these oscillations were significantly attenuated following 6-HOF treatment. Notably, ROS and calcium oscillations act in concert during osteoclastogenesis [58], and 6-HOF suppressed both processes, suggesting that it may limit osteoclast overactivation and functional enhancement by disrupting the ROS–Ca²⁺ feedback loop.In the in vivo experiments, an LPS-induced calvarial osteolysis mouse model was employed to further confirm the anti-resorptive effects of 6-HOF. Micro-CT three-dimensional reconstruction revealed severe calvarial erosion and a significant reduction in bone volume fraction (BV/TV) following LPS stimulation, whereas 6-HOF administration markedly ameliorated these pathological changes. Quantitative bone morphometric analysis demonstrated that6-HOF effectively restored BV/TV levels and reduced bone surface porosity, indicating that it can suppress inflammation-mediated bone destruction in vivo. These findings are consistent with the in vitro results, further validating the pharmacological activity and biological feasibility of 6-HOF in mitigating inflammatory osteolysis.

Building on the elucidation of the molecular mechanisms by which 6-HOF regulates osteoclast differentiation and function, evaluation of its pharmacokinetic properties is essential for assessing its translational relevance. Although the BOILED-Egg model does not directly predict bone tissue distribution, it provides informative insight into the systemic exposure potential of candidate compounds following oral administration. In this study, BOILED-Egg analysis predicted favorable gastrointestinal absorption of 6-HOF (WLOGP = 3.17, TPSA = 50.44 Å²), supporting its potential development as an orally available small-molecule agent. Currently, the clinical management of inflammatory osteolysis and osteoclast-related disorders relies mainly on bisphosphonates and the RANKL monoclonal antibody denosumab [59, 60]. However, despite their proven efficacy, long-term bisphosphonate therapy is associated with gastrointestinal adverse effects, atypical fractures, and osteonecrosis of the jaw, while denosumab requires parenteral administration and carries a risk of rebound bone loss after treatment discontinuation [61, 62]. These limitations highlight the ongoing need for alternative therapeutic strategies with improved safety profiles and greater convenience.

In this context, small molecules with favorable oral absorption and the ability to systemically modulate osteoclast activity remain of considerable interest. Although 6-HOF has not been engineered for bone-targeted delivery, it markedly suppresses osteoclast differentiation and bone-resorptive function under inflammatory conditions by attenuating oxidative stress, normalizing aberrant calcium oscillations, and activating the Nrf2 signaling pathway. These findings suggest that 6-HOF may exert bone-protective effects within inflammation-driven bone microenvironments even in the absence of direct bone targeting. Nevertheless, the present study is primarily based on a classical inflammatory osteolysis model, and direct evidence regarding the in vivo pharmacokinetics and bone tissue distribution of 6-HOF remains limited. Future studies integrating pharmacokinetic profiling, tissue distribution analyses, and more complex disease models will be necessary to further define and optimize its translational potential.

## Conclusions

In summary, 6-HOF inhibits inflammatory osteolysis by activating the Keap1/Nrf2 antioxidant pathway and suppressing Ca²⁺–NFATc1 signaling, thereby reducing oxidative stress and osteoclast activity.These findings highlight its therapeutic potential against inflammation-induced bone destruction.

## Supplementary Information

Supplementary Material 1 (XLSX 102 KB)

Supplementary Material 2 (CSV 11.1 KB)

Supplementary Material 3 (XLSX 35.4 KB)

Supplementary Material 4 ( XLSX 9.88 KB)

Supplementary Material 5 (XLSX 6.46 KB)

Supplementary Material 6 (XLSX 102 KB)

## Data Availability

The data supporting the findings of this study are available from the corresponding author upon reasonable request.
